# A global genomic approach uncovers novel components for twitching motility-mediated biofilm expansion in *Pseudomonas aeruginosa*

**DOI:** 10.1099/mgen.0.000229

**Published:** 2018-11-01

**Authors:** Laura M. Nolan, Cynthia B. Whitchurch, Lars Barquist, Marilyn Katrib, Christine J. Boinett, Matthew Mayho, David Goulding, Ian G. Charles, Alain Filloux, Julian Parkhill, Amy K. Cain

**Affiliations:** ^1^​ MRC Centre for Molecular Bacteriology and Infection (CMBI), Department of Life Sciences, Imperial College London, London SW7 2AZ, UK; ^2^​ The ithree Institute, University of Technology Sydney, Ultimo, NSW 2007, Australia; ^3^​ Institute for Molecular Infection Biology, University of Würzburg, Würzburg D-97080, Germany; ^4^​ Helmholtz Institute for RNA-based Infection Research (HIRI), Würzburg, Germany; ^5^​ Wellcome Sanger Institute, Wellcome Genome Campus, Hinxton, Cambridge, UK; ^6^​ Quadram Institute of Bioscience, Norwich Research Park, Norwich, Norfolk NR4 7UA, UK; ^†^​ Present address: Hospital for Tropical Diseases, Wellcome Trust Major Overseas Programme, Oxford University Clinical Research Unit, Ho Chi Minh City, Vietnam.; ^‡^​ Present address: Chemical and Biomolecular Sciences, Macquarie University, Sydney, NSW, Australia.

**Keywords:** T4P, *Pseudomonas aeruginosa*, biofilm, twitching motility

## Abstract

*Pseudomonas aeruginosa* is an extremely successful pathogen able to cause both acute and chronic infections in a range of hosts, utilizing a diverse arsenal of cell-associated and secreted virulence factors. A major cell-associated virulence factor, the Type IV pilus (T4P), is required for epithelial cell adherence and mediates a form of surface translocation termed twitching motility, which is necessary to establish a mature biofilm and actively expand these biofilms. *P. aeruginosa* twitching motility-mediated biofilm expansion is a coordinated, multicellular behaviour, allowing cells to rapidly colonize surfaces, including implanted medical devices. Although at least 44 proteins are known to be involved in the biogenesis, assembly and regulation of the T4P, with additional regulatory components and pathways implicated, it is unclear how these components and pathways interact to control these processes. In the current study, we used a global genomics-based random-mutagenesis technique, transposon directed insertion-site sequencing (TraDIS), coupled with a physical segregation approach, to identify all genes implicated in twitching motility-mediated biofilm expansion in *P. aeruginosa*. Our approach allowed identification of both known and novel genes, providing new insight into the complex molecular network that regulates this process in *P. aeruginosa*. Additionally, our data suggest that the flagellum-associated gene products have a differential effect on twitching motility, based on whether components are intra- or extracellular. Overall the success of our TraDIS approach supports the use of this global genomic technique for investigating virulence genes in bacterial pathogens.

## Data Summary

All supporting data and protocols have been provided within the article or through supplementary data files. Four supplementary tables and three supplementary figures are available with the online version of this article. All sequences from the transposon directed insertion-site sequencing (TraDIS) assays are available in the European Nucleotide Archive (ENA) under study accession number ERP001977. Individual ENA accession numbers of each sample are ERS427191–3 for the non-twitching cells, ERS427194–6 for the twitching cells and ERS427197–9 for the base library without selection.

**Impact Statement**

Treatment of bacterial infections is becoming increasing challenging due to rising levels of antibiotic resistance, particularly for high-risk pathogens. *Pseudomonas aeruginosa* is a leading cause of hospital-borne infections and causes chronic infections in cystic fibrosis patients. One major infection strategy of this bacterium is the formation of protective biofilms. *P. aeruginosa* biofilms can rapidly spread between host tissues, as well as along the length of implanted medical devices, such as catheters, using a form of bacterial motion called twitching motility. Despite some understanding of the genetic basis of twitching motility, the complex regulatory pathways and multiple components involved mean a systematic molecular approach is needed to gain a complete understanding of this important process. The current work is significant as it uses a powerful, high-throughput approach to discover the full set of genes involved in twitching motility, providing key insights into how *P. aeruginosa* infections can establish and spread.

## Introduction

*Pseudomonas aeruginosa* is a leading cause of healthcare-associated infections and is the major cause of mortality in patients with cystic fibrosis (CF) [1]. This bacterium’s success as a pathogen is mainly attributed to its ability to produce a plethora of cell-associated and secreted virulence factors [2]. Type IV pili (T4P) are major cell-associated virulence factors of *P. aeruginosa*, promoting both attachment to host epithelial cells and a form of flagella-independent surface translocation, termed twitching motility [3]. Twitching motility is a complex and co-ordinated multicellular phenomenon, which in *P. aeruginosa* results in active biofilm expansion [4, 5]. This active expansion can result in the spread of infection within host tissues and along implanted medical devices [6, 7].

The biogenesis, assembly and regulation of the T4P for mediating twitching motility-mediated biofilm expansion requires at least 44 different proteins [3, 4, 8]. The components involved in biogenesis and assembly of T4P are encoded by *pilA*, *B*, *C*, *D*, *E*, *F*, *M*, *N*, *O*, *P*, *Q*, *T*, *U*, *V*, *W*, *X*, *Y1*, *Y2* and *Z*, and *fimT*, *U* and *V*. The T4P is composed of multiple PilA monomers, which are assembled by the T4P biogenesis machinery. This machinery is composed of the motor, alignment and pilus subcomplexes [3]. At the inner membrane, the motor subcomplex is composed of a platform protein PilC and the cytoplasmic ATPases PilB and PilT, which are responsible for pilus elongation and retraction, respectively [9, 10]. The alignment subcomplex composed of PilM, PilN and PilO forms a connection between the motor subcomplex and the outer membrane-associated secretin complex of PilP and PilQ [11–14]. Pilus extension is mediated by the combined activity of PilZ, FimX and the ATPase PilB, while the ATPases PilT and/or PilU are responsible for pilus retraction [15–21].

Expression of these T4P genes is controlled by several systems in *P. aeruginosa*, including the two-component sensor–regulator pairs, PilS/PilR [22, 23] and FimS/AlgR [24]. The highly complex Chp chemosensory system encoded by the *pilGHIJK–chpABC* gene cluster [25–28] is involved in regulating the motors which control T4P extension and retraction in response to environmental signals [29], and is related to the Che chemosensory signal transduction system in *Escherichia coli* which regulates flagella-mediated chemotaxis [30]. Other regulatory components include: the virulence factor regulator Vfr [homologous to the *E. coli* catabolic repressor protein (CRP)] [31, 32]; FimL, which appears to intersect with the Chp chemosensory system and Vfr regulatory cascade [33]; Crc, the catabolic repressor control protein [34]; FimX [21, 35]; FimV [36]; as well as PocA, PocB and TonB3 [37, 38]. Additionally, the regulation of T4P biogenesis, assembly and twitching motility-mediated biofilm expansion is further complicated by the contribution of the small intracellular signalling molecules 3′,5′-cyclic adenosine monophosphate (cAMP) and 3′,5′-cyclic diguanylic acid (c-di-GMP) [35, 39, 40]. Clearly the regulation of T4P biogenesis and assembly, and twitching motility-mediated biofilm expansion is complex, and while multiple components and signalling cascades have been implicated, it remains unclear precisely how these components and pathways intersect. Therefore, we predict that other, currently uncharacterized proteins and pathways provide the links between these known components and pathways that regulate T4P biogenesis, assembly and twitching motility.

Here we adapted a global genomics-based approach termed transposon directed insertion-site sequencing (TraDIS) [41, 42] to identify all genes involved in twitching motility-mediated biofilm expansion in *P. aeruginosa*. TraDIS is a powerful method which utilizes high-throughput sequencing of dense random transposon mutant libraries to identify all genes involved in any selective condition [42, 43]. TraDIS (and related transposon-insertion sequencing methods) has proved successful for assaying a number of phenotypes in *P. aeruginosa*, including antibiotic resistance [44], stress conditions [45] and *in vivo* wound infection [46]. However, this is the first time TraDIS has been used to investigate biofilm expansion in *P. aeruginosa.* The success of our systematic and global approach for investigating this aspect of *P. aeruginosa* virulence demonstrates that TraDIS-based separation is a powerful method to generate a comprehensive catalogue of genes involved in motility and pathogenesis-associated phenotypes, and the physical segregation approach used here can be applied more broadly to study bacterial phenotypes other than simple survival in selective conditions.

## Methods

### Bacterial strains, media and twitching motility assays

Strains used in this study were *P. aeruginosa* strain PA14, mutants from the PA14 non-redundant transposon mutant library [47] (see Table S2, available in the online version of this article) and *E. coli* S17-1 containing the mini-Tn*5*-pro plasmid [48]. *P. aeruginosa* strain PAK (Filloux lab collection) was used for generation of mutant strain PAK_05353 (orthologue of PA5037/PA14_66580) generated by allelic exchange mutagenesis as described previously [49, 50]. Additionally, PA14*pilR *:: mar2xT7 [47] was used as a pilin-negative control for transmission electron microscopy (TEM) analysis and twitching motility subsurface stab assays, and PAKΔ*pilQ* (gift from Stephen Lory) and PAK*pilA *: TcR [51] as negative controls for Western blot analyses.

*P. aeruginosa* and *E. coli* were cultured on Lysogeny broth (LB) [52] solidified with agar at 1.5 % (w/v) or 1 % (w/v) (for twitching motility subsurface assays) and grown overnight at 37 °C. Cultures were grown in either cation-adjusted Mueller Hinton broth (CAMHB) or LB, and incubated overnight at 37 °C, with shaking at 250 r.p.m. Gentamicin at 15 µg ml^−1^ was used for plasmid maintenance in *E. coli* and for recovery of PA14 transposon mutants from library glycerol stocks; after initial recovery of PA14 transposon mutants from glycerol no antibiotic selection was used. Twitching motility-mediated biofilm expansion was assayed in subsurface stab assays as described previously [40]. The generated TraDIS library transposon mutants were recovered on 1× Vogel-Bonner Media (VBM) [a 10× solution contains MgSO_4_.7H_2_O (8 mM), citric acid (anhydrous) (9.6 mM), K_2_HPO_4_ (1.7 mM), NaNH_5_PO_4_.4H_2_O (22.7 mM), pH 7, and filter sterilized] with 1.5 % (w/v) agar containing gentamycin at 100 µg ml^−1^.

### Planktonic growth assays

Planktonic growth of *P. aeruginosa* was followed by recording changes in OD_600nm_ for 20 h, with incubation at 37 °C and shaking at 250 r.p.m. as described previously [53]. Cells were grown in 96-well microtitre plates with LB to replicate the conditions in the subsurface twitching motility assays, or minimal media (1× M63) [(NH_4_)_2_SO_4_ (15 mM); KH_2_PO_4_ (22 mM) and K_2_HPO_4_ (40 mM)] supplemented with MgSO_4_ (1 mM), casamino acids (0.05 %, w/v) and glucose (0.4 %, w/v) to replicate the conditions in the attachment assays.

### Submerged biofilm assays

Overnight cultures were diluted to an OD_600nm_ of 0.1 into microtitre plates with 1× M63 media plus supplements (as for minimal media growth assays above) and incubated statically at 37 °C for 18 h. Planktonic growth was then removed and the remaining attached cells were stained with crystal violet [10 % (v/v)] for at least 10 min, statically, at room temperature. Unbound crystal violet stain was removed and the plate was washed twice prior to extraction of crystal violet dye with ethanol [95 % (v/v)]. OD_600nm_ of the crystal violet dye was then used to quantify the levels of attached cells.

### DNA manipulation

DNA isolation was performed using the PureLink Genomic DNA mini kit (Life Technologies) except for TraDIS library genomic DNA isolation (see below). Isolation of plasmid DNA was carried out using the QIAprep spin miniprep kit (Qiagen). Primers (Sigma) used are shown in Table 1. DNA fragments were amplified with either KOD Hot Start DNA Polymerase (Novagen) or standard Taq polymerase (NEB) as described by the manufacturer with the inclusion of Betaine (Sigma) or DMSO (Sigma). Restriction endonucleases were used according to the manufacturer’s specifications (Roche). DNA sequencing was performed by GATC Biotech.

**Table 1.** Oligonucleotides used in this study

**Name**	**Oligonucleotide 5′−3′ sequence**	**Description**
PA5037_1	GCTGGCACTGGAGCCGACCG	For deletion mutagenesis of PAK*05353* (*PA14_66580*) in PAK/PAO1 - primer 1
PA5037_2	TCAATGCGCGCTGGTCATGGGACCTCA	For deletion mutagenesis of PAK*05353* (*PA14_66580*) in PAK/PAO1 - primer 2
PA5037_3	ATGACCAGCGCGCATTGATCCGACCTG	For deletion mutagenesis of PAK*05353* (*PA14_66580*) in PAK/PAO1 - primer 3
PA5037_4	CGGCTTTCACCGGGTCCTGG	For deletion mutagenesis of PAK*05353* (*PA14_66580*) in PAK/PAO1 - primer 4
PA5037_5	CCTGGGCGGCGGCGTCATC	For deletion mutagenesis of PAK*05353* (*PA14_66580*) in PAK/PAO1 - primer 5
PA5037_6	ATAGAAGGCGGCCAGGTCGGC	For deletion mutagenesis of PAK*05353* (*PA14_66580*) in PAK/PAO1 - primer 6

### Preparation of samples for PilQ immunoblotting

Preparation of whole cell samples for PilQ analysis was performed as described previously [28] with cells being harvested from plates grown for 20 h at 37 °C on LB agar. For analysis of PilQ multimerization, samples were only boiled for 2 min at 95 °C in Laemmli loading buffer prior to loading on the SDS-PAGE gel. All other Western blot samples were boiled for 10 min at 95 °C in Laemmli loading buffer prior to loading on the SDS-PAGE gel.

### Western blot analysis

SDS-PAGE and Western blotting were performed as described previously [50]. Proteins were resolved in 8, 10, 12 or 15 % gels using the Mini-PROTEAN system (Bio-Rad) and transferred to nitrocellulose membrane (GE Healthcare) by electrophoresis. Membranes were blocked in skimmed milk [5 % (w/v)] (Sigma) before incubation with primary antibodies. Membranes were washed with TBST [0.14 M NaCl, 0.03 M KCl and 0.01 M phosphate buffer plus Tween 20 (0.05 %, v/v)] before incubation with horesradish peroxidase (HRP)-conjugated secondary antibodies (Sigma). The resolved proteins on the membrane blots were detected using the Novex ECL HRP Chemioluminescent substrate (Invitrogen) or the Luminata Forte Western HRP substrate (Millipore) using a Las3000 Fuji Imager. Membranes were probed with α-PilQ antibody (gift from Stephen Lory), or α-RNAP antibody (Neoclone) and secondary anti-rabbit antibody for PilQ and anti-mouse antibody for RNAP.

### Transmission electron microscopy assays

Log phase cultures (OD_600nm_ of 0.25–0.5) were fixed in the planktonic state with 0.1 % glutaraldehyde and then spotted on a 400 mesh copper/palladium grid. Alternatively, cells were first spotted on a grid, incubated for 15 min at room temperature, and then fixed in a surface-associated state with 0.1 % (v/v) glutaraldehyde. Preparations were then washed three times with water and negatively stained twice with 1 % (v/v) uranyl acetate. Images were taken with an FEI Morgagni 268(D) electron microscope.

### TraDIS library generation

A highly saturated transposon mutant library was generated in *P. aeruginosa* PA14 by large-scale conjugation with an *E. coli* SM17-1 (mini-Tn*5*-pro) donor, which allowed for random insertion of a mariner transposon throughout the PA14 genome, and conferred gentamicin resistance in the recipient PA14 strain. The *E. coli* donor strain was grown in LB supplemented with gentamicin (15 µg ml^−1^) overnight at 37 °C and the recipient PA14 strain was grown overnight at 37 °C in CAMHB. Equivalent amounts of both strains were spread uniformly on separate LB agar plates and incubated overnight at 37 °C for *E. coli* and at 43 °C under humid conditions for *P. aeruginosa*. The next day, one *E. coli* donor plate was harvested and combined by extensive physical mixing on a fresh LB agar plate with one plate of harvested recipient PA14 strain. Conjugation between the two strains was achieved by incubation of the high-density mixture of both strains at 37 °C for 2 h. The conjugation mix was then harvested, pelleted by centrifugation (10 000 ***g***, 10 min, 4 °C), and resuspended in LB. The resuspended cells were recovered on 1× VBM agar supplemented with gentamicin (100 µg ml^−1^) and incubated for 17 h at 37 °C. The numbers of mutants obtained were estimated by counting a representative number of colonies across multiple plates. Mutants on plates were recovered as a pool, resuspended in LB, pelleted by centrifugation (10 000 ***g***, 10 min, 4 °C), and then resuspended in LB plus glycerol [15 % (v/v)] and stored at −80 °C. The protocol was repeated on a large scale until ~2 million mutants were obtained.

### TraDIS assay with mutant pool

The transposon mutant library pool was diluted 1 : 10 into 9 ml CAMHB in 10 separate 50 ml Falcon tubes which were covered with aeroseal to facilitate aeration within the culture and incubated at 37 °C overnight. Thirty millilitres of LB agar [1.5 % (w/v)] 90 mm plates was poured and allowed to set overnight at room temperature. The following morning the agar was flipped into a larger Petri dish to expose the smooth underside set against the Petri dish base, which promotes rapid twitching motility-mediated biofilm expansion [54]. Then, 1.5 ml of overnight growth of the pooled transposon mutant library was pelleted by centrifugation (10 000 ***g*** , 3 min, 4 °C), and the whole pellet was spotted into the centre of the flipped agar plate. This was repeated for all 10 overnight cultures and performed in triplicate (i.e. a total of 30 plates). All plates were incubated under humid conditions at 37 °C for 65 h. To harvest mutants based upon their ability to undergo twitching motility-mediated biofilm expansion, mutants were harvested from the inner, non-twitching zone and from the outer, active-twitching motility zone (see Fig. S1a) for all three replicates. The cells from the inner and from the outer zones were harvested separately by scraping cells with a 10 µl inoculating loop and resuspended in 5 ml LB, followed by cell pelleting by centrifugation (10 000 ***g***, 10 min, 4 °C), for all three replicates. The supernatant was discarded and the cells were used for genomic DNA extraction.

### Genomic DNA extraction for TraDIS library sequencing

Genomic DNA from the harvested pooled library pellets was resuspended in 1.2 ml lysis solution [Tris-HCl (10 mM), NaCl (400 mM) and Na_2_EDTA (2 mM)], supplemented with proteinase K in storage buffer [Tris-HCl (50 mM), glycerol (50 %, v/v), NaCl (100 mM), EDTA (0.1 mM), CaCl_2_ (10 mM), Triton X-100 (0.1 %, w/v) and DTT (1 mM)] to a concentration of 166 µg ml^−1^. Cell lysis was achieved by incubation at 65 °C for 1 h, with occasional vortexing. The samples were then cooled to room temperature and RNA was removed by addition of RNaseA (5 µg ml^−1^) and incubation at 37 °C for 80 min. Samples were then placed on ice for 5 min. Each lysate was then split into two Eppendorf tubes each of ~600 µl, and 500 µl NaCl (3 M) was added to each tube. Cell debris was removed by centrifugation (10 000 ***g***, 10 min, 4 °C) and 500 µl from each tube was added to two volumes of isopropanol to precipitate DNA. DNA was then collected by centrifugation (10 000 ***g***, 10 min, 4 °C), with the pelleted DNA being washed twice in 70 % (v/v) ethanol. DNA was finally resuspended in 50 µl Tris-EDTA buffer.

### Generation of DNA sequencing libraries and library sequencing

TraDIS was performed using the method described by Barquist *et al*. [42]. The PCR primers used were designed in this study: for library construction (5′ AATGATACGGCGA CCACCGAGATCTACACAGGTTGAACTGCCAACGACT ACG and 3′ AATGATACGGCGACCACCGAGATCTACACAACTCTCTACTGTTTCTCCATACCCG) and sequencing TraDIS primers (5′ CGCTAGGCGGCCAGATCTGAT and 3′ GGCTAGGCCGCGGCCGCACTTGTGTA); during library amplification, plasmid block primers were used to prevent amplification of plasmid background (5′ ctagaagaagcttgggatccgtcgaccgatcccgtacacaagtagcgtcc–dideoxy and 3′ attccaca aattgttatccgctcacaattccacatgtggaattccacatgtgg-dideoxy). For the twitching motility TraDIS sequencing, we used a MiSeq Illumina platform and 13.2 million 150 bp single-end sequencing reads were generated. Reads were mapped onto the PA14 (accession number: CP000438) genome, 10 % of the 3′ end of each gene was discounted, and a 10 read minimum cut-off was used in comparisons performed using EdgeR [55], using scripts from the Bio-TraDIS pipeline ([42]; https://github.com/sanger-pathogens/Bio-Tradis). All sequences from the TraDIS assays are available in the European Nucleotide Archive (ENA) under study accession number ERP001977 and individual ENA accessions of each sample are ERS427191–3 for the non-twitching cells, ERS427194–6 for the twitching cells and ERS427197–9 for the base library without selection.

### Downstream analysis of TraDIS results

KEGG enrichment analysis was performed in R. KEGG pathway annotations were retrieved using the KEGGREST package (v1.6.0 10.18129/B9.bioc.KEGGREST; https://bioconductor.org/packages/3.7/bioc/html/KEGGREST.html). A hypergeometric test was used to test for pathway enrichment in genes with higher (logFC>4, q-value<0.01) or lower (logFC<−4, q-value<0.01) mutant abundance in the TraDIS assay, as reported in Tables S3 and S4.

## Results

### Confirmation of genes known to be involved in twitching motility

To identify genes involved in twitching motility-mediated biofilm expansion we generated a high-density random transposon mutant library in *P. aeruginosa* PA14 using conjugation of a Tn*5* minipro vector and gentamicin selection. We determined that this library consisted of 310 000 unique Tn*5* mutants by sequencing DNA from 10^9^ cells from the raw base library, in duplicate, without selection.

Approximately 10^9^ cells from an overnight culture of the pool of transposon mutants were concentrated and inoculated as a central spot on top of an inverted agar plate. These were incubated for 65 h at 37 °C under humid conditions to allow a twitching motility-mediated surface biofilm to form. An inverted agar plate was used to expose the smooth underside of the moist, set agar, which facilitates rapid twitching motility-mediated biofilm expansion and discourages other forms of motility [54]. This colony biofilm assay was favoured over the subsurface twitching motility assay [54] as it allows a much greater number of cells to be recovered, thus allowing sufficient amounts of genomic DNA to be extracted for downstream sequencing. Transposon mutants were separated based upon their ability to expand via twitching motility, away from the site of inoculation, with cells being harvested from the inner, non-twitching section of the colony biofilm, and the outer, actively expanding edge (Fig. S1a). The outer and inner zones from 10 plates were combined to form each replicate, and three replicates were performed over different days. Genomic DNA was extracted from both combined pools of mutants and then separately sequenced to determine the number of insertions per gene, using a TraDIS approach, as described previously [42]. The relative frequencies of transposon insertion in the non-twitching and twitching transposon mutant pools were compared as described previously [42] and using a cut off of log_2_FC=4 and a q-value of <0.01 to identify genes with differential insertion levels during twitching motility-mediated biofilm expansion. This revealed 942 genes as having a putative role in twitching motility-mediated biofilm formation: 82 genes with increased insertions and 860 with decreased insertions (Table S1). Forty-two of the 44 genes known to be involved in twitching motility-mediated biofilm expansion were identified [3, 4, 8] (Table 2). The two additional known genes that could not by assayed by our TraDIS screen were *rpoN* (PA14_57940) and *pocB* (PA14_25500), as they did not contain transposon insertions in the input PA14 base library.

**Table 2.** TraDIS identification of genes known to be involved in twitching motility

**Gene name**	**PA14 orthologue**	**Gene product**	TraDIS log-fold change*
*algR*	PA14_69470	alginate biosynthesis regulatory protein AlgR	−10.21
*chpA*	PA14_05390	ChpA	−12.74
*chpB*	PA14_05400	ChpB	+7.82
*chpC*	PA14_05410	putative chemotaxis protein methyltransferase CheR	−1.08†
*crc*	PA14_70390	catabolite repression control protein	−12.78
*fabF1*	PA14_25690	beta-ketoacyl-acyl carrier protein synthase II	−3.82
*fimL*	PA14_40960	pilin biosynthetic protein	−10.25
*algZ*	PA14_69480	alginate biosynthesis protein AlgZ/FimS	−6.79
*fimT*	PA14_60270.1	type 4 fimbrial biogenesis protein FimT	−1.24†
*fimU*	PA14_60280	type 4 fimbrial biogenesis protein FimU	−12.53
*fimV*	PA14_20860	putative T4P pilus assembly protein FimV	+1.04
*fimX*	PA14_65540	conserved hypothetical protein	−6.89
*pocB*	PA14_25500	conserved hypothetical protein	na
*pilA*	PA14_58730	type IV pilin structural subunit	+0.86†
*pilB*	PA14_58750	type 4 fimbrial biogenesis protein PilB	−8.85
*pilC*	PA14_58760	type 4 fimbrial biogenesis protein pilC	−10.62
*pilD*	PA14_58770	type 4 prepilin peptidase PilD	−6.46
*pilE*	PA14_60320	type 4 fimbrial biogenesis protein PilE	−9.70
*pilF*	PA14_14850	type 4 fimbrial biogenesis protein PilF	−5.29
*pilG*	PA14_05320	type IV pili response regulator PilG	−8.64
*pilH*	PA14_05330	type IV pilus response regulator PilH	−3.17
*pilI*	PA14_05340	type IV pili signal transduction protein PilI	−10.18
*pilJ*	PA14_05360	type IV pili methyl-accepting chemotaxis protein PilJ	−7.73
*pilK*	PA14_05380	methyltransferase PilK	−0.18†
*pilM*	PA14_66660	type 4 fimbrial biogenesis protein PilM	−6.22
*pilN*	PA14_66650	type 4 fimbrial biogenesis protein PilN	−6.86
*pilO*	PA14_66640	type 4 fimbrial biogenesis protein PilO	−11.35
*pilP*	PA14_66630	type 4 fimbrial biogenesis protein PilP	−6.96
*pilQ*	PA14_66620	type 4 fimbrial biogenesis OM protein PilQ precursor	−6.20
*pilR*	PA14_60260	two-component response regulator PilR	−12.27
*pilS*	PA14_60250	kinase sensor protein of two component regulatory	−6.48
*pilT*	PA14_05180	twitching motility protein PilT	−9.69
*pilU*	PA14_05190	twitching motility protein PilU	−8.54
*pilV*	PA14_60280.1	type 4 fimbrial biogenesis protein PilV	−13.14
*pilW*	PA14_60290	type 4 fimbrial biogenesis protein PilW	−8.71
*pilX*	PA14_60300	type 4 fimbrial biogenesis protein PilX	−8.86
*pilY1*	PA14_60310	type 4 fimbrial biogenesis protein PilY1	−7.56
*pilY2*	PA14_60310.1	type 4 fimbrial biogenesis protein PilY2	−9.50
*pilZ*	PA14_25770	type 4 fimbrial biogenesis protein PilZ	−9.03
*ppk*	PA14_69230	polyphosphate kinase	+3.94
*rpoN*	PA14_57940	RNA polymerase sigma-54 factor	na
*rpoS*	PA14_17480	sigma factor RpoS	−5.85
*tonB3*	PA14_05300	TonB3	−7.53
*vfr*	PA14_08370	cyclic AMP receptor-like protein	−9.30

*A positive log-fold change indicates that the gene product has a negative effect on twitching motility, while a negative log-fold change indicates a positive effect on twitching motility.

†Result is not significant (q-value>0.05) but confirmed by visual inspection to have differential insertion levels during twitching motility-mediated biofilm expansion; na, not assayed due to a minimal insertion density in these genes in our starting base library.

### Identification of novel components involved in twitching motility-mediated biofilm expansion

From our TraDIS results we selected 39 genes that had not been previously implicated in twitching motility (Table S2) for phenotypic characterization using single transposon mutants from the non-redundant PA14 transposon mutant collection [47]. We tested the ability of each mutant to undergo twitching motility using a subsurface stab assay. For those target genes that had multiple transposon mutants available, we tested all mutants, bringing the total number of assayed mutants to 52 (Table S2). From these assays, we detected 32 transposon mutants which had significantly altered levels of twitching motility compared to the wild-type (Fig. S2).

**Fig. 1.**
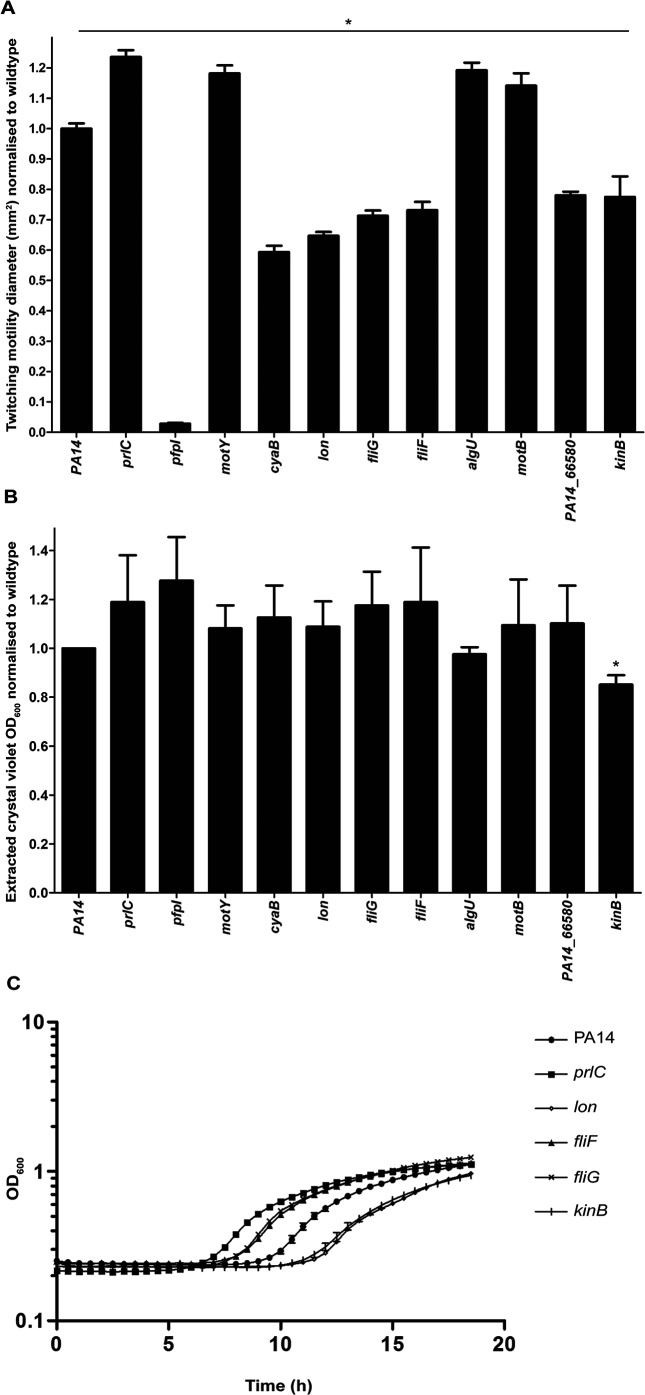
Characterization of twitching motility, biofilm and growth phenotypes for selected transposon mutants. (a) Subsurface twitching motility-mediated interstitial biofilm expansion at agar/plastic interface after 48 h of incubation at 37 °C is presented as the mean (±sem) surface area (mm^2^) normalized against the wild-type as obtained from two independent experiments performed in triplicate. A two-tailed Student’s *t*-test was used; *P<0.0005 compared to wild-type. (b) Submerged biofilm formation in 96-well microtitre plates after 18 h at 37 °C presented as the mean (±sem) OD_600nm_ of the mean of extracted crystal violet staining normalized against the wild-type, from four independent experiments performed in triplicate. A two-tailed Student’s *t*-test was used; *P<0.005 compared to wild-type. (c) Growth rates determined by incubation of transposon mutants at 37 °C for 19 h in LB media. Mean OD_600_ values for *prlC*, *lon*, *fliF, fliG* and *kinB* at each time point were significantly different (*P*<0.05) from the wild-type, as predicted by a one-way ANOVA with Dunnett’s multiple comparison test, and the mean OD_600_ values for *kinB, cyaB, pfpI, motB, motY, algU* and *PA14_66580* at each time point were not significantly different from PA14 wild-type.

We selected 11 transposon mutants for further characterization based on biological interest and especially dramatic changes in twitching ability (Table 3). The genes containing these transposon insertions appear to group into distinct functional classes including cellular metabolism, signal transduction, cytokinesis and flagella-mediated motility (Table 3). Biofilm and growth assays were conducted for these 11 selected mutants to determine whether there was any effect on submerged biofilm formation (Fig. 1b) and also to determine if the observed twitching motility defect (Fig. 1a) was due to a growth-related effect (Fig. 1c). Of these only *kinB* was found to have decreased levels of biofilm formation compared to the wild-type (Fig. 1b), as reported previously [56]. None of these transposon mutants had an altered ability to grow in the minimal medium used for the biofilm assay, demonstrating that any alteration in biofilm formation was not a result of a growth-related effect (Fig. S1b). Of these 11 transposon mutants, *prlC*, *lon*, *kinB*, *fliF* and *fliG* had significant alterations in growth rate in LB media (the same media used for subsurface twitching motility assays) compared to the wild type (Fig. 1c). Specifically, *fliF*, *fliG* and *prlC* had a shorter lag time than the wild-type, and *kinB* and *lon* had a longer lag time, but all reached approximately the same final cell density (Fig. 1c). Based on these results, a growth-related effect may account for some of the observed decrease in twitching motility for *kinB* and *lon*. However, the observed alterations in twitching motility for *prlC* (increased twitching motility), *PA14_66580* (decreased twitching motility)*, pfpI* (decreased twitching motility)*, fliG* (decreased twitching motility)*, motY* (increased twitching motility) and *algU* (increased twitching motility) are unlikely to be solely accounted for by growth-related defects. Of these AlgU has already been implicated in regulation of twitching motility via what appears to be an indirect mechanism [24]. To our knowledge none of the remaining targets, *prlC*, *PA14_66580*, *pfpI*, *fliG* and *motY*, have been previously implicated in twitching motility in *P. aeruginosa*. Examination of the 3348 strains available on Pseudomonas.com [57] (database version 17.2) revealed that there are over 500 orthologous gene matches for each of these genes: *prlC* (*n*=557); *PA14_66580* (*n*=557); *pfpI* (*n*=1262); *fliG* (*n*=561); and *motY* (*n*=557), demonstrating that all of these genes are highly conserved across the genus *Pseudomonas*. To confirm that the observed twitching motility phenotypes were a consequence of transposon disruption of the target genes and not altered expression of downstream genes in the operon, subsurface stab assays were conducted for transposon mutants of genes in each of the operons containing *prlC*, *pfpI*, *fliG* and *motY* (Fig. S3). These genes were: *PA14_00810* (downstream of *prlC*), *PA14_04640* (downstream of *pfpI*), *PA14_50080*, *PA14_50100*, *PA14_50110* (downstream of *fliG*) and *PA14_18740* (downstream of *motY*). Where there was more than one transposon mutant available in the PA14 mutant collection [47], the most N-terminal transposon insertion mutant was selected. The stab assays revealed that the twitching motility phenotype of the downstream gene mutants was not significantly different from the wild-type, and thus a polar effect of the transposon insertion on downstream gene expression does not account for the observed phenotype. Note that transposon mutants of genes downstream of PA14_65580 were not assayed here as a clean deletion mutant was generated for further analysis (see below).

**Table 3.** Transposon mutants with increased or decreased twitching motility compared to PA14 wild-type for further analysis

PA14 transposon mutant*	**Twitching motility phenotype relative to wild-type**	**PAO1 orthologue**	**Functional class**†	**Description**
PAMr_nr_mas_07_1 : C3	Increased	*prlC*	Amino acid transport and metabolism	Zn-dependent oligopeptidase
PAMr_nr_mas_04_1 : A9	Decreased	*lon*	Post-translational modification, protein turnover, chaperones	ATP-dependent Lon protease
PAMr_nr_mas_07_1 : D4	Decreased	*kinB*	Signal transduction mechanisms	Signal transduction histidine kinase
PAMr_nr_mas_12_4 : D5	Decreased	*cyaB*	Signal transduction mechanisms	Adenylate cyclase
PAMr_nr_mas_12_1 : C6	Decreased	*pfpI*	Bacterial-type flagellar swarming motility; cellular response to antibiotic; single-species biofilm formation	Intracellular protease
PAMr_nr_mas_05_1 : H3	Decreased	*fliG*	Cell motility	Flagellar motor switch protein
PAMr_nr_mas_09_4 : G3	Decreased	*fliF*	Cell motility, intracellular trafficking, secretion and vesicular transport	Flagellar basal body M-ring protein
PAMr_nr_mas_07_1 : C7	Increased	*motB*	Bacterial-type flagellar cell motility	Flagellar motor protein
PAMr_nr_mas_10_2 : F2	Increased	*motY*	Cell wall/membrane/envelope biogenesis	Sodium-dependent flagellar system protein
PAMr_nr_mas_09_3 : C3	Increased	*algU*	Sigma factor activity; negative regulation of bacterial-type flagellar cell motility; regulation of polysaccharide biosynthetic process	Sigma factor
PAMr_nr_mas_14_4 : C8	Decreased	*PA5037* (*PA14_66580*)	ATPase activity; Type II secretory pathway, component ExeA	Hypothetical

*Plate and well for mutants from PA14 transposon mutant collection (Liberati *et al.* [47]).

†Colours denote mutants which have similar predicted functional classes for the gene product using Clusters of Orthologous Groups (COGs).

### Visualizing the T4P of novel twitching motility gene targets using TEM

**Fig. 2.**
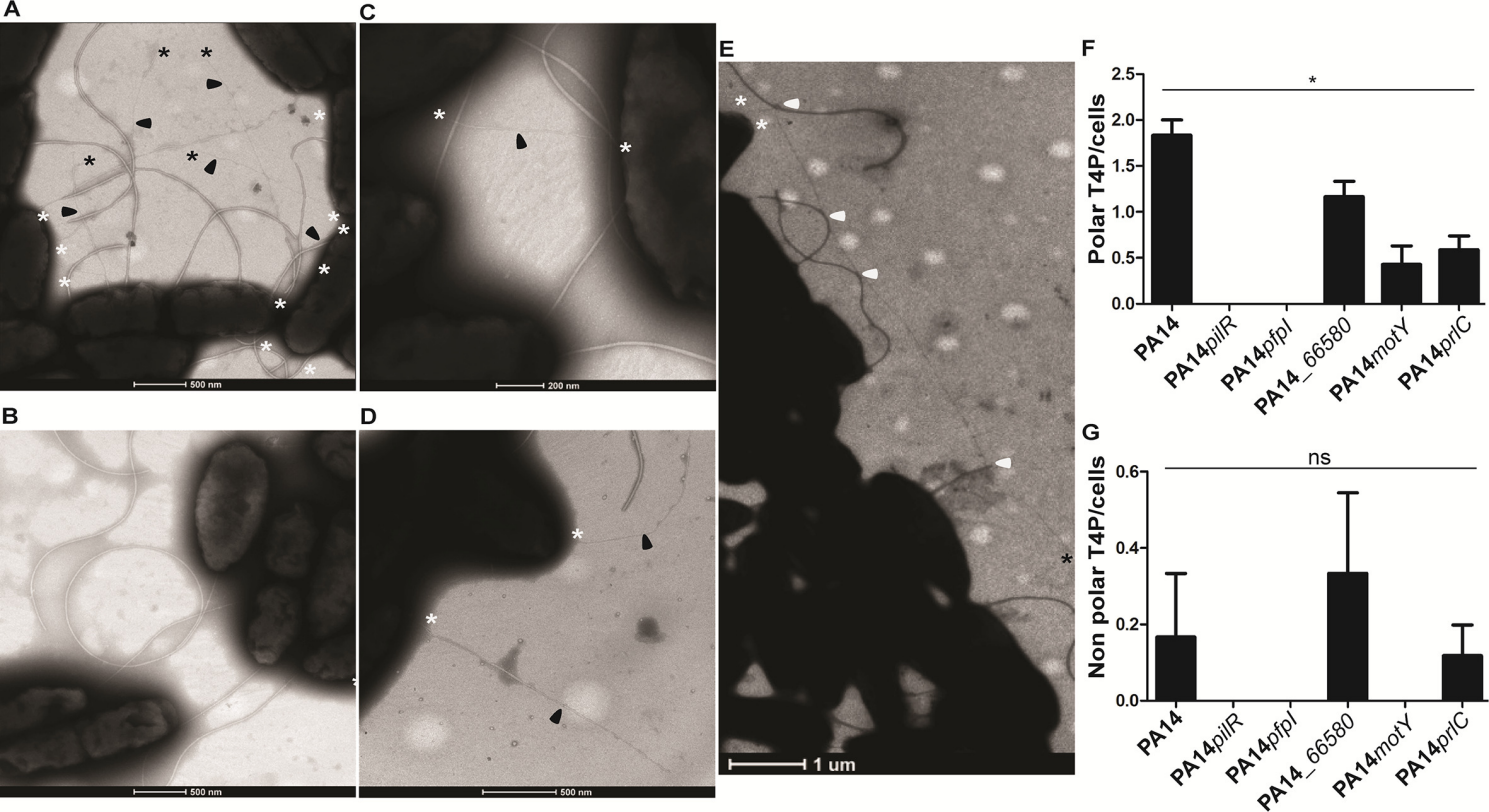
Visualization of T4P assembly and localization. Representative images of (a) wild-type PA14, (b) a pili mutant *pilR*, (c) a *PA14_66580* mutant, (d) a *motY* mutant and (e) a *prlC* mutant are shown; (f, g) quantification of T4P at the polar or non-polar cellular region/cells. All pili visualized and included in our analyses were 1–2 µm in length. *pfpI* cells were indistinguishable from *pilR* as represented in (b); a *prlC* mutant had longer T4P compared to the wild-type and in some cases these pili appeared to interact with the flagella (potential interactions marked with white arrows) (e). In each image the pili are arrowed in black, with a black asterisk at any free ends and a white asterisk where the pili appear to join or go under a cell membrane. Images are representative of triplicate grids imaged in biological triplicate. For each replicate of each strain at least 200 cells were visualized. In (f) a two-tailed Student’s *t*-test was used; *P<0.05 compared to the wild-type; in (g) a two-tailed Student’s *t*-test was used; ns determined for all samples compared to the wild-type: versus PA14*pilR P*=0.363, versus PA14*pfpI P*=0.361, versus *PA14_66580* *P*=0.611, versus PA14*motY P*=0.363, versus PA14*prlC P*=1.000.

Alterations in twitching motility levels are commonly attributed to an increase or decrease in levels of expressed and/or assembled T4P and/or mislocalization of T4P. To determine whether the alterations in twitching motility of certain mutants result from abnormal levels or mislocalization of T4P, TEM was used to visualize the pili. We investigated *prlC*, *PA14_66580*, *pfpI* and *motY*, as flagellum-related representatives, as well as PA14 wild-type and *pilR*, as respective positive and negative controls (Fig. 2). These experiments revealed that PA14 wild-type, *PA14_66850*, *motY* and *prlC* possess pili which were mainly polar (Fig. 2a, c–g). Overall, the non-twitching *pfpI* mutant (Fig. 1a) had no observable T4P (Fig. 2b), both *PA14_66850* and *motY* had a reduced number of polar pili compared to the wild-type (Fig. 2c, d, f) and the hyper-twitching mutant *prlC* (Fig. 1a) had extra-long pili, which in some cases appeared to intertwine with the observed flagella (Fig. 2e) and an overall reduction in polar T4P levels compared to the wild-type (Fig. 2f). While PA14 wild-type, *PA14_66850* and *prlC* were found to possess non-polar T4P in a few cases, there was no difference in the numbers of non-polar T4P in the mutant strains compared to the wild-type (Fig. 2g).

### Investigating the role of *PA14_66580* in T4P assembly and function

**Fig. 3.**
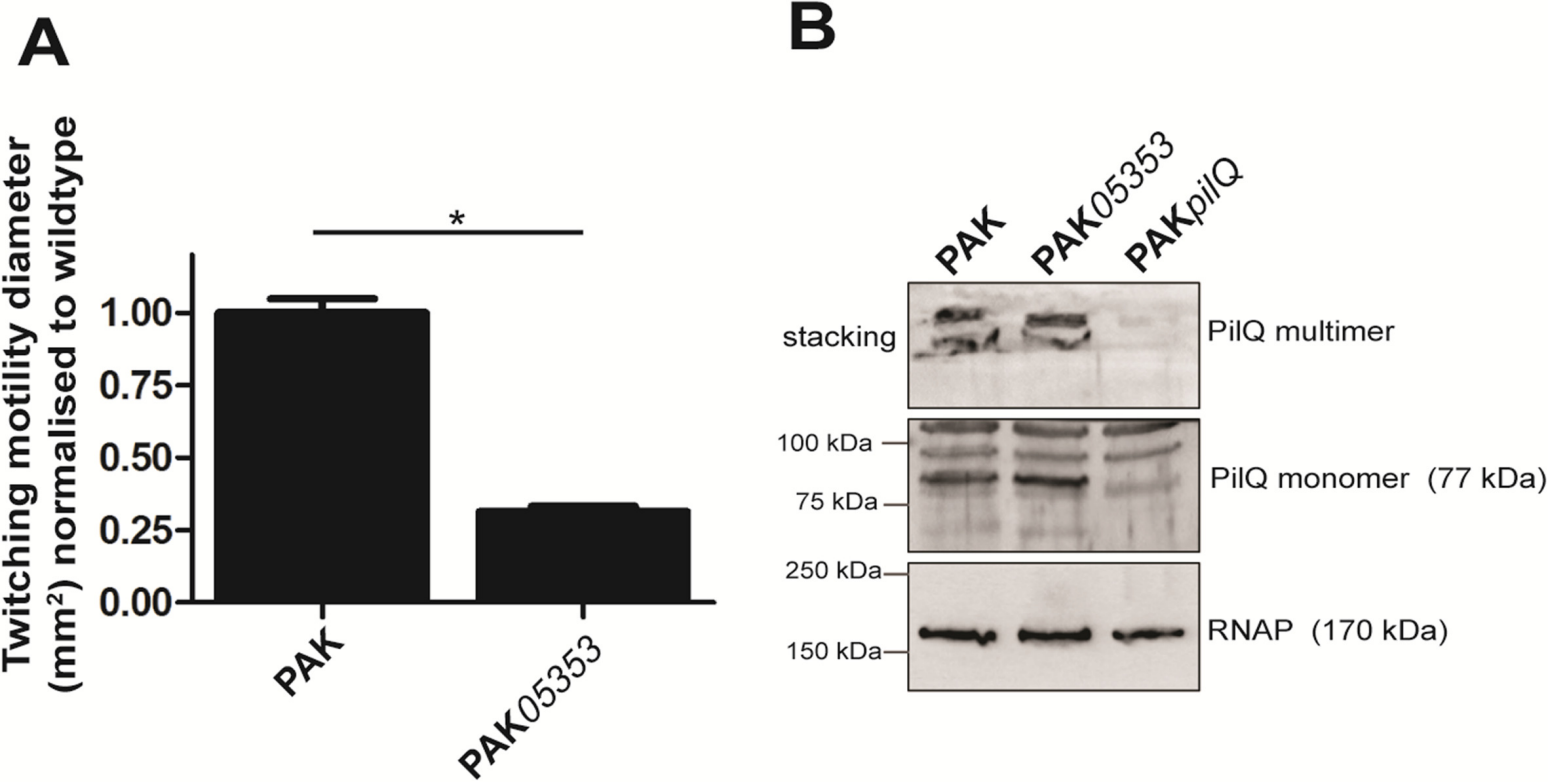
Twitching motility and PilQ secretin phenotypes in PAK*05353* (*PA14_66580* mutant). (a) Subsurface twitching motility-mediated interstitial biofilm expansion at agar/plastic interface after 48 h of incubation at 37 °C for PAK and clean deletion of *PA14_66580* in PAK (PAK*05353*) is presented as the mean (±sem) surface area (mm^2^) normalized against the wild-type as obtained from three independent experiments performed in triplicate. A two-tailed Student’s *t*-test was used; *P<0.0005 compared to the wild-type. (b) Immunoblot of PilQ from whole cell preparations of strains PAK, PAK*05353* and PAK*pilQ* obtained from overnight (20 h) confluent lawns grown at 37 °C on LB agar plates. RNAP was used as a loading control.

A clean deletion in *PA14_66580* was generated in the orthologous gene (*PAK_05353* with the gene product having 99.82 % amino acid identity to PA14_66580) in the *P. aeruginosa* strain PAK. As was observed for the transposon mutant of *PA14_66580* in PA14, a reduction in twitching motility was also observed in the PAK deletion mutant PAK*05353* (Fig. 3a). *PA14_66580*/*PAK_05353* is encoded just upstream of the *pilMNOP* gene cluster, which encodes the components in the alignment subcomplex, and the outer membrane-associated secretin complex of PilP and PilQ, which is involved in T4P outer membrane extrusion [11–14]. Additionally, PA14_66580/PAK_05353 is also annotated as a predicted ExeA-like protein. ExeA is an ATPase that binds peptidoglycan and is involved in transport and multimerization of ExeD into the outer membrane to form the functional secretin of the Type II secretion system [58]. Given this, we hypothesized that PA14_66580/PAK_05353 may be involved in multimerization and/or localization of the PilQ secretin complex. To investigate this we performed immunoblotting of whole cell lysates of wild-type, PAK*05353* and PAK*pilQ* strains harvested from agar plates for both the multimeric and monomeric forms of PilQ (Fig. 3b). This revealed that PAK*05353* was able to form both multimers and monomers of PilQ to the same extent as the wild-type, indicating that PAK05353 does not appear to play a role in PilQ multimerization.

### Functional gene enrichment analysis

Enrichment analyses of genes that had increased or decreased mutant populations in the TraDIS output using the KEGG database [59] revealed that three key pathways were significantly altered: flagella biosynthesis, two-component systems (TCS) and chemotaxis [Table S3 (increased population) and Table S4 (decreased population)].

**Fig. 4.**
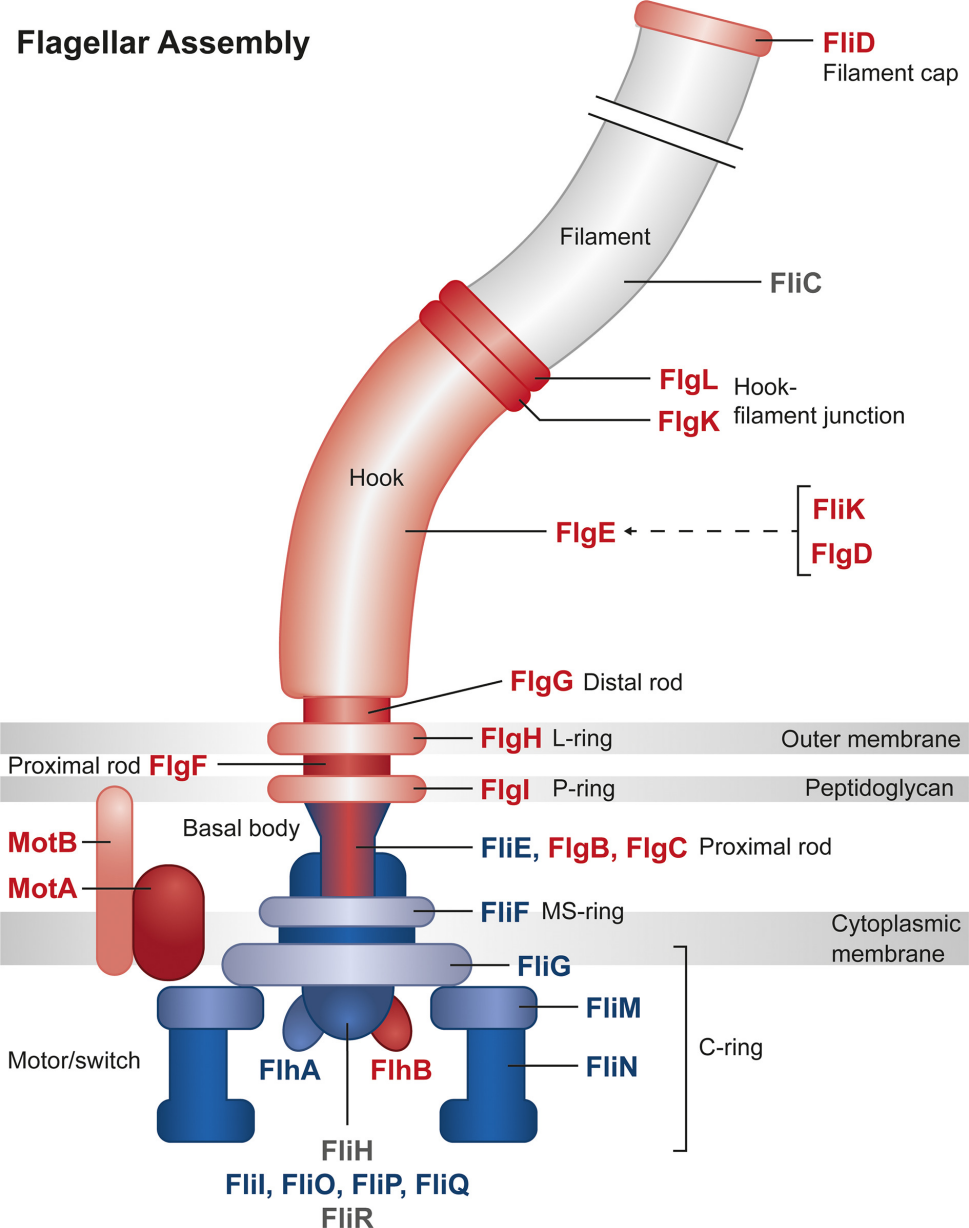
Relative log-fold change of transposon insertions in genes for flagella components. Gene products involved in flagella structure or regulation of flagella function are represented in this diagram. Genes which had a positive log fold change (which implies a negative effect on twitching motility) are coloured red, and are mostly located in the outer part of the cell envelope and flagellum body. Genes which had a negative log fold change (which implies a positive effect on twitching motility) are coloured blue and are mostly located in the inner part of the cell envelope and flagellum body. Output image generated from the KEGG Mapper tool (http://www.kegg.jp/kegg/mapper.html).

We noted that a number of flagella-associated structural and regulatory genes had altered mutant abundances following selection for twitching motility-mediated biofilm formation in our TraDIS assay, and some single mutants were confirmed to have significantly altered levels of twitching motility compared to the wild-type (Fig. 1a). Remarkably, this revealed a strong correlation between gene products predicted to have a negative effect on twitching motility [which corresponds to a positive log-fold change in our TraDIS output (Table S1), or a measured increase in twitching motility of the transposon mutant (Fig. S2)] with proteins associated with the outer part of the cell envelope and thus the outer part of the flagellum body. In contrast, proteins associated with the inner part of the cell envelope and flagellum body were predicted to have a positive effect on twitching motility [which corresponds to a negative log-fold change in our TraDIS output (Table S1) or a measured decrease in twitching motility of the transposon mutant (Fig. S2)] (Fig. 4).

The chemotaxis pathway identified in our functional gene enrichment analysis included mutants of swimming chemotaxis (*che*) genes which appeared to promote (*cheA/B/Z/Y*) as well as inhibit (*cheR/W*) twitching motility-mediated biofilm formation. This suggests a balance between bacterial chemotaxis and twitching motility, especially as the chemotaxis pathway also controls flagella assembly. The TCS linked to twitching motility were mostly known genes, for instance *algZ/R* involved in alginate biosynthesis, or the *pil* genes in T4P production, but also included some unexpected genes related to osmotic stability, such as *cusS/R* involved in copper efflux, or *dctA/B/D/P* for C4-dicarboxtrate transport.

## Discussion

In this study, we have successfully applied a physical separation-based TraDIS approach to identify genes involved in twitching motility-mediated biofilm formation in *P. aeruginosa*. Using this method, we detected almost all genes currently known to be involved in T4P assembly and twitching motility, in addition to a large number of genes identified in our TraDIS output (Table S1) and a select group for further study (Table 3) not previously known to be involved.

A functional enrichment analysis of all genes that have altered mutant abundances in our assay identified three major groups of gene function that were affected during twitching motility: flagella assembly, bacterial chemotaxis and TCS. Perhaps the most interesting from these is the potential involvement of the flagella as suggested from the predicted (Table S1) or determined (Fig. S2) differential effect of structural and regulatory flagella components on twitching motility. Specifically, we observed a strong correlation between gene products predicted to have a negative effect on twitching motility with proteins associated with the outer part of the flagella body, and in contrast, proteins associated with the inner part of the flagella body were predicted to have a positive effect on twitching motility (Fig. 4). This is intriguing as it suggests a differential effect on twitching motility by flagella components based upon their cellular location and certainly warrants further investigation in future work.

For each of the 11 gene targets selected for further investigation (Table 3) the twitching motility phenotype was confirmed in a subsurface stab assay, with growth assays performed to demonstrate that the observed twitching phenotype was not solely accounted for by a growth defect. Submerged biofilm formation was also assayed and revealed that only *kinB* had a significant decrease in levels compared to the wild-type (Fig. 1b), demonstrating that our TraDIS assay did indeed selectively identify genes specific for twitching motility-mediated biofilm expansion on a semi-solid surface. Overall these assays confirmed that the twitching motility phenotype observed for *prlC*, *PA14_66580*, *pfpI*, *fliG* and *motY* was not solely due to a growth-related defect. For these mutants TEM was used to determine whether the twitching motility phenotype could be attributed to alterations in levels and/or localization of surface assembled T4P. No pili were observed in a *pfpI* mutant (Fig. 2b), which explains the observed lack of twitching motility (Fig. 1a). PfpI is an intracellular protease which affects antibiotic resistance, swarming motility and biofilm formation in *P. aeruginosa* [60]; however, to our knowledge the current study is the first to report a role for PfpI in twitching motility. Given the established role of intracellular proteases in controlling levels of a range of chaperones and regulatory proteins it is likely that the protease activity of PfpI is required for control of regulators or other proteins involved in T4P biogenesis and/or assembly.

A role for PA14_66580 was also investigated in the formation of the PilQ secretin to allow T4P extrusion and thus function. This was based upon the proximity of *PA14_66580* to the *pilMNOP* operon, which encodes components that link the outer membrane PilQ secretin to the inner membrane motor complex. Additionally PA14_66580 possesses the same conserved domain as ExeA (Uniprot: http://www.uniprot.org/uniprot/A0A0H2ZID1), which is an ATPase that binds peptidoglycan and is involved in transport and multimerization of ExeD into the outer membrane to form the secretin of the Type II secretion system [58]. Our TEM data revealed that *PA14_66580* had reduced numbers of pili compared to the wild-type (Fig. 2c, f), which correlates with the observed reduction in twitching motility in both PA14 and PAK strain backgrounds (Figs 1a and 3a). Given that no difference in the expression of monomeric or multimeric PilQ was observed in a mutant of *PA14_566580* in PAK (PAK*05353*) (Fig. 3b), we suggest that the reduction in surface T4P and twitching motility levels is not due to a lack of secretin formation. PA14_66580 could instead be involved in stabilization of the secretin pore and/or formation of the assembly and motor subcomplexes in order to allow full functionality of the T4P.

A *prlC* mutant was found to have increased levels of twitching motility compared to the wild-type (Fig. 1a), a reduction in polar surface assembled T4P (Fig. 2f) and to have a putative interaction between the surface-assembled flagella and T4P (Fig. 2d). PrlC is uncharacterized in *P. aeruginosa*, although it has an M3 peptidase domain (Pfam PF01432) which is associated with mammalian and bacterial oligopeptidases. The homologue in *E. coli* is a cytoplasmic protease (also named PrlC) which appears to be a partner in degradation of peptides produced by ATP-dependent proteases from multiple protein degradation pathways [61]. A homologue of PrlC also exists in *Aeromonas hydrophilia*. A mutant of the oligopeptidase *pepF* was shown to have decreased swimming motility, increased biofilm formation in a crystal violet microtitre plate assay and increased attachment to epithelial cells [62]. While the exact role of PepF has been not elucidated, these published data suggest that PepF could be involved in processing proteins involved in biogenesis or regulation of the polar flagella, used for swimming, or the bundle-forming pili (Bfp) or Type-IV *Aeromonas* pili (Tap) used for attachment. While it is unclear exactly how PrlC in *P. aeruginosa* influences twitching motility, our results suggest a similar role as for PepF in *A. hydrophilia* in processing proteins involved in biogenesis, assembly or regulation of the T4P. Alternatively, given the putative interaction of the flagella and T4P observed (Fig. 2d), PrlC may be involved in processing flagella-associated proteins to ultimately affect the putative interaction between these two motility machines and thus the function of the T4P (as suggested from Fig. 4).

We observed that a *motY* mutant had increased twitching motility levels (Fig. 1a) but reduced levels of T4P (Fig. 2a, f). MotY is a peptidoglycan binding protein which is required for MotAB-mediated flagella motor rotation and is associated with the outer-membrane [63]. While a *motY* mutant is severely impaired for flagella-mediated motility on semi-solid surfaces (swarming motility), there is only a slight decrease in levels of swimming motility compared to the wild-type in liquid media [63]. The *motY* mutant used in this study [47] has a transposon insertion in the centre of the protein, and thus lacks the C terminus of the protein, which has been shown to be involved in stabilizing the association of MotY with the stator proteins MotAB to allow flagella rotation [64]. Given the observed reduction in surface assembled pili (Fig. 2a, f) and increase in twitching motility (Fig. 1a) this suggests that, as discussed above, a defect in flagella function is likely to be affecting the function of the T4P to produce the observed twitching motility phenotype.

FliG is one of the proteins in the rotor-mounted switch complex (C ring), located at the base of the basal body in the cytoplasm, and is important for directing flagella rotation [30]. We observed a decrease in twitching motility of *fliG* (Fig. 1a). Deletion of the C-terminal region of FliG (as is the case for our *fliG* transposon mutant [47]) results in a strain which produces non-functional flagella [65]. Thus we would predict that, as for *motY*, our *fliG* mutant would also have wild-type levels of assembled T4P, with the presence of non-functional flagella in *fliG* also potentially affecting function of the T4P in twitching motility.

This study has identified both known and novel components involved in twitching motility in *P. aeruginosa*. Additionally, we have provided analyses which suggest a differential effect of flagella proteins on T4P function based upon their cellular location and points towards a possible interaction between the flagella and T4P machinery to influence twitching motility. Overall these results highlight the success of our TraDIS-based approach and point to a number of intriguing new players involved in twitching motility-mediated biofilm expansion in *P. aeruginosa*.

## Data bibliography

1. Lee DG, Urbach JM, Wu G, Liberati NT, Feinbaum RL *et al*. Genomic analysis reveals that *Pseudomonas aeruginosa* virulence is combinatorial. Genome biology. 2006;7 (10):R90. Reports the genome sequence of *P. aeruginosa* PA14 used as reference sequence in the current study.
2. Nolan LM, Whitchurch CB, Barquist L, Katrib M, Boinett CJ *et al*. All transposon directed insertion-site sequencing (TraDIS) assay data with ENA study accession number ERP001977 (2018).
3. Nolan LM, Whitchurch CB, Barquist L, Katrib M, Boinett CJ *et al*. ENA for non-twitching transposon mutant population: ERS427191-3 (2018).
4. Nolan LM, Whitchurch CB, Barquist L, Katrib M, Boinett CJ *et al*. ENA for twitching transposon mutant population: ERS427194-6 (2018).
5. Nolan LM, Whitchurch CB, Barquist L, Katrib M, Boinett CJ *et al*. ENA for the base library without selection: ERS427197-9 (2018).

## Supplementary Data

Supplementary File 1

Supplementary File 2
