# Supplementary material for: A global genomic approach uncovers novel components for twitching motility-mediated biofilm expansion in Pseudomonas aeruginosa
Source: Microb Genom. 2018 Nov 1;4(11):e000229. doi: 10.1099/mgen.0.000229 (PMC6321873; doi:10.1099/mgen.0.000229)
Supplement: Supplementary File 1 [file mgen-4-229-s001.pdf]

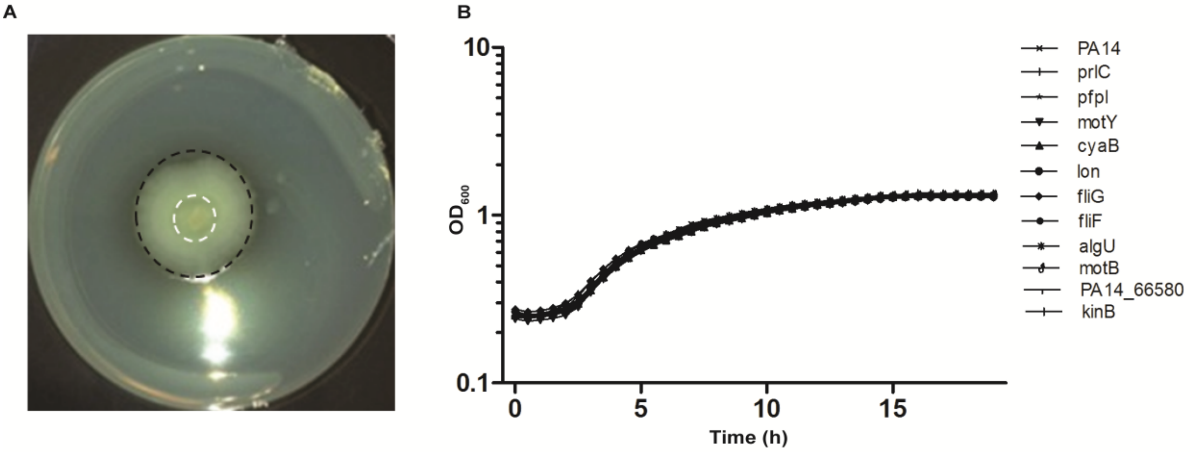

Figure S1. (A) The transposon mutant pool was inoculated in the centre of an inverted agar plate and incubated for 72 h at 37 °C. Mutants were separated based upon their ability to undergo twitching motility-mediated biofilm expansion away from the inoculation site. After 72 hrs mutants were harvested from the inner non-motile region (outlined in white) and the outer active twitching edge (outlined in black). Genomic DNA was extracted from each pool of mutants and sequenced using a mass parallel approach. **(B)** Growth rates in minimal media for 11 selected transposon mutants assayed for a submerged biofilm defect in Figure 1B. Growth rates were determined by incubation of transposon mutants at 37 °C for 19 h in M63 minimal media (same media used for submerged biofilm assays). There was no significant different between growth rates of transposon mutants compared to wildtype predicted by a one-way ANOVA with Dunnett's multiple comparison test. Data are represented as the mean ± standard deviation for 2 independent replicates performed in triplicate.

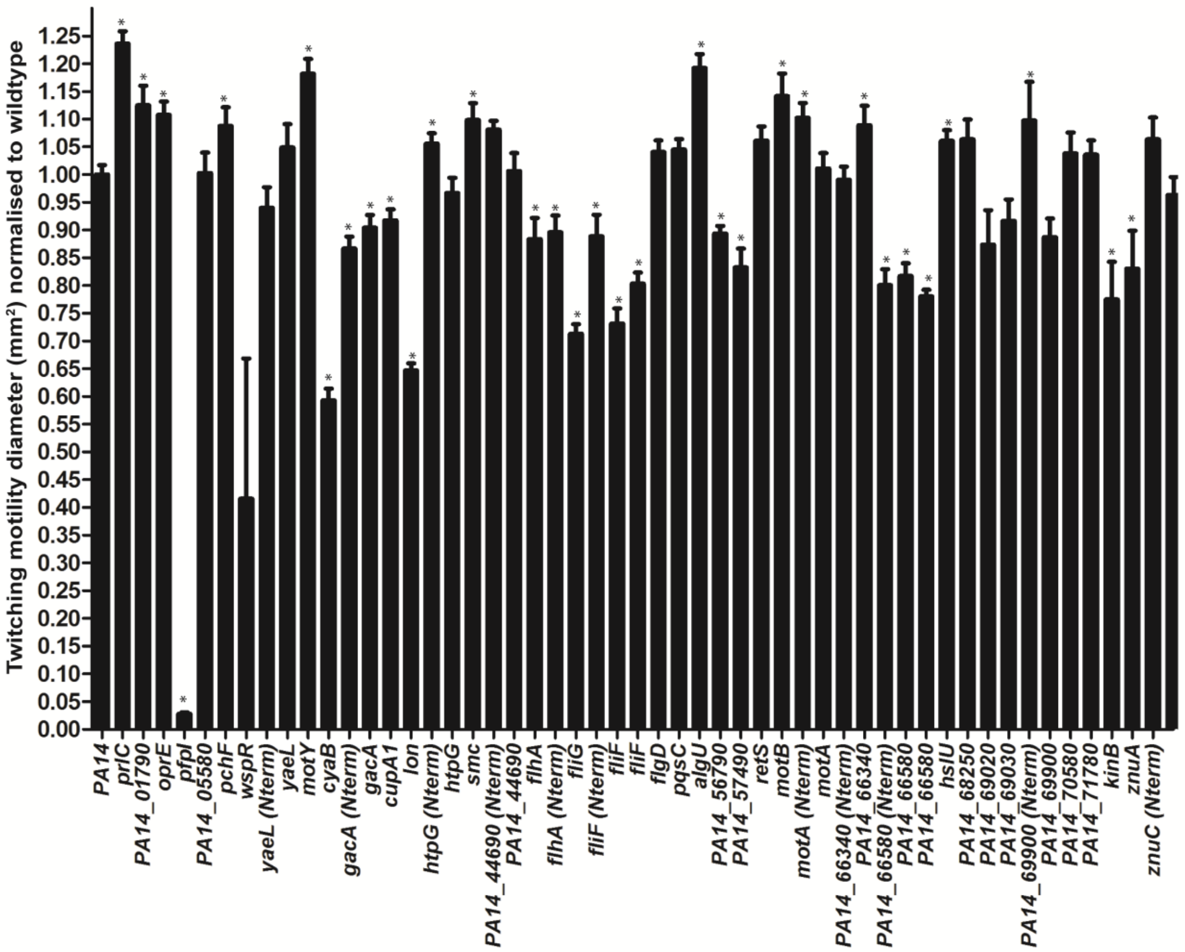

Figure S2. Twitching motility of selected transposon mutant targets identified using TraDIS. Sub-surface twitching motility-mediated interstitial biofilm expansion at agar/plastic interface after 48 h incubation at 37 °C is presented as the average surface area in mm^2 ± standard deviation normalized against wildtype as obtained from 2 independent experiments performed in triplicate. Two-tailed student's t-test, * ($p<0.005$) compared to wildtype.

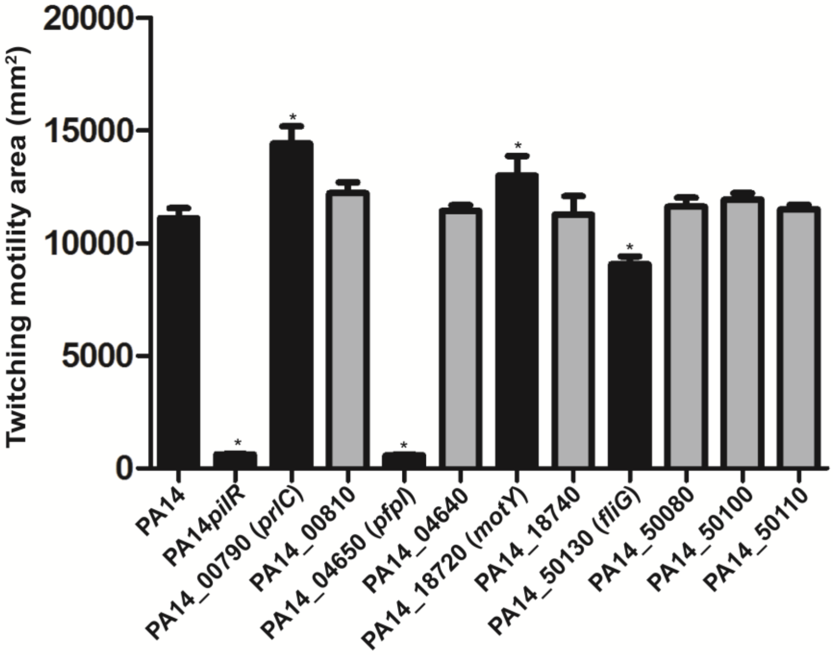

Figure S3. Twitching motility of transposon mutants in genes downstream of *prlC, pfpI, fliG* and *motY*. Sub-surface twitching motility-mediated interstitial biofilm expansion at agar/plastic interface after 48 h incubation at 37 °C is presented as the mean surface area in mm^2 ± standard error of the mean as obtained from 2 independent experiments performed in triplicate. *prlC, pfpI, fliG and motY* are black bars and the transposon mutants in the operon downstream of each of these genes are grey bars. Two-tailed student's t-test, * ($p<0.05$) compared to wildtype; ns for *PA14_00810* (downstream of *prlC*) (p = 0.098) compared to wildtype; ns for *PA14_04640* (downstream of *pfpI*) (p = 0.546) compared to wildtype; ns for *PA14_50080* (p = 0.437) or *PA14_50100* (p = 0.381) or *PA14_50110* (p = 0.147) (all downstream of *fliG*) compared to wildtype; and ns for *PA14_18740* (downstream of *motY*) (p = 08.28) compared to wildtype.
